# Oral lipid prodrug V2043 (1-*O*-octadecyl-2-*O*-benzyl-*sn*-glyceryl-P-remdesivir nucleoside) protects mice from lethal dengue infection

**DOI:** 10.1128/aac.00401-26

**Published:** 2026-08-03

**Authors:** Alex E. Clark, Jonathan R. Erlich, James R. Beadle, Rachel E. McMillan, Jialei Xie, Aaron F. Garretson, Shae Atkins, Anoushka Sharma, Suraj Manohar Rajan, Ben A. Croker, Robert T. Schooley, Karl Y. Hostetler, Aaron F. Carlin

**Affiliations:** 1 Department of Pathology, University of California San Diego, La Jolla, California, USA; 2 Department of Medicine, University of California San Diego, La Jolla, California, USA; 3 Department of Pediatrics, University of California San Diego, La Jolla, California, USA; 4 Department of Infectious Diseases, J. Craig Venter Institute, La Jolla, California, USA

**Keywords:** remdesivir nucleoside, Japanese encephalitis virus, Zika virus, West Nile virus, mouse model, *in vivo* efficacy, broad spectrum antiviral, remdesivir, lipid prodrug, dengue

## Abstract

Dengue virus and related flaviviruses are significant threats to global health, with no approved antiviral treatments available. Remdesivir triphosphate (RVn-TP) is a nucleotide analog that inhibits RNA-dependent RNA polymerases from many clinically important RNA viruses, including dengue virus. An orally bioavailable prodrug that efficiently delivers RVn-TP could facilitate early treatment for non-hospitalized dengue patients. To that end, we developed 1-*O*-octadecyl-2-*O*-benzyl-*sn*-glyceryl-P-remdesivir nucleoside (V2043, ODBG-P-RVn), a phospholipid prodrug that achieves RVn-TP delivery through improved oral bioavailability, potency, and pharmacokinetics. Here, we show that when compared to the parent compound remdesivir nucleoside (RVn), V2043 is a more potent *in vitro* inhibitor of multiple flaviviruses, including dengue, Zika, West Nile, and Japanese encephalitis viruses. In lethal prophylactic and therapeutic mouse models that recapitulate key aspects of severe dengue in humans, once-daily oral V2043 significantly decreased dengue serum viremia, clinical severity, weight loss, and mortality. Additionally, oral V2043 was well tolerated when administered daily for 14 days. These results demonstrate the potential therapeutic efficacy of V2043 to treat several clinically relevant flaviviruses, including dengue virus, the most prevalent and widespread arbovirus infection in the world.

Development of antiviral therapies to treat patients infected with dengue virus (DENV) is a critical health priority. Cases of dengue have dramatically increased in recent years, with over 14 million reported cases and 12,000 deaths worldwide in 2024 (1). Currently, there are no antivirals for DENV, and vaccination efforts are complicated by the risk of worsening disease outcomes, likely due to antibody-dependent enhancement (ADE) in the context of sub-neutralizing antibody titers (2). Remdesivir (GS-5734, Veklury) is a prodrug of remdesivir nucleoside (RVn) monophosphate (RVn-MP) that is broadly effective against many RNA viruses, including flaviviruses (3, 4). It has a high barrier to resistance and is FDA-approved for the treatment of COVID-19. GS-5743 is more potent than RVn due to its ProTide design that enhances cell entry and increases the rate of metabolism to RVn-TP by bypassing rate-limiting initial phosphorylation (5). However, the clinical use of GS-5734 has been limited by the requirement for intravenous administration due to poor oral pharmacokinetics and short plasma half-life (5, 6). An orally bioavailable prodrug that produces high tissue levels of RVn-TP through novel prodrug design would enable outpatient administration and create potential treatment options for dengue and other RNA viruses with no currently available therapies.

Lipid prodrugs can improve drug oral bioavailability, pharmacokinetics, and potency. We developed a lipid RVn prodrug 1-*O*-octadecyl-2-*O*-benzyl-*sn*-glyceryl-P-RVn (ODBG-P-RVn, V2043), which is efficiently absorbed in large quantities by enterocytes (7–9). Consequently, V2043 is absorbed by the gastrointestinal tract and circulates intact, resulting in excellent bioavailability, 73.6%, and a long plasma half-life (7, 10). V2043 maintains potent broad-spectrum antiviral activity, likely due to rapid cell entry and direct metabolism to RVn-MP without requiring initial rate-limiting phosphorylation, similar to GS-5734 (10). *In vivo*, oral V2043 significantly reduced SARS-CoV-2 lung titers in both prophylaxis and treatment mouse models (7, 10, 11). Thus, the lipid prodrug design of V2043 achieves excellent oral bioavailability, pharmacokinetics, and broad-spectrum antiviral potency that suggests its utility against other RNA viruses.

In this study, we evaluated the *in vitro* activity of V2043 against multiple clinically important flaviviruses and *in vivo* efficacy in a lethal dengue virus mouse model. We found that V2043 is more potent than RVn *in vitro* against dengue (DENV) serotypes 1–4, Zika virus (ZIKV), West Nile virus (WNV), and Japanese encephalitis virus (JEV) in human hepatocytes and primary monocyte-derived macrophages. *In vivo*, daily oral treatment of infected interferon-alpha/beta- and -gamma-receptor-deficient AG129 mice significantly reduced serum viral loads and was well tolerated. Protection against disease in infected mice was measured in a prophylactic pretreatment model, a delayed treatment model, and a dose-reduction model by evaluating survival, body weight change, and clinical scores up to 10 days post-infection. We found that once-daily oral V2043 treatment of 80 mg/kg with a 160 mg/kg loading dose increased survival and reduced disease severity when treatment was started up to 2 days post-infection and was 100% protective if started 1 day after infection. V2043 maintained protection against disease and death when initiated at the time of infection at one-half and one-quarter of the initial dosing regimen. Overall, these data support the therapeutic potential of V2043 to treat dengue and other related flaviviruses that pose a threat to human health.

## RESULTS

### V2043 is a more potent flavivirus inhibitor compared to RVn

**FIG 1:**
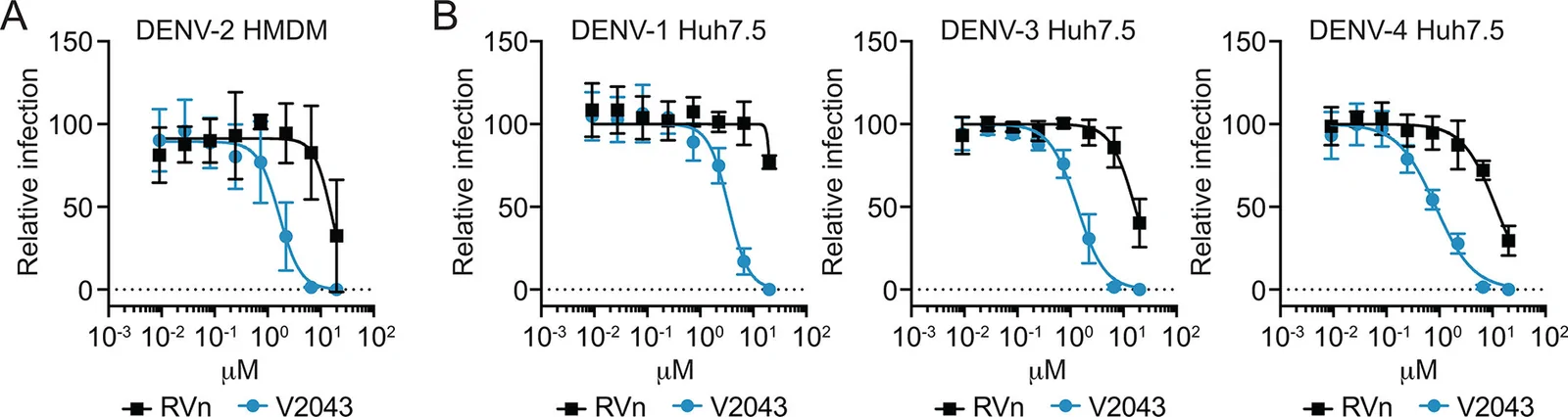
*In vitro* antiviral activity of V2043 and RVn against flaviviruses. (A) Primary human monocyte-derived macrophages (HMDMs) were pretreated with compounds for 30–60 min and infected with DENV-2 UIS 353 at an MOI of 1 FFU/cell (based on initial plating density) for 18 h. Viruses were pre-incubated 1 h before infection with human IgG-anti-flavivirus antigen (4G2) to mediate antibody-dependent enhancement (ADE) of infection. (B) Huh7.5 cells were pretreated with compounds for 30–60 min and infected with DENV-1, DENV-3, or DENV-4 at an MOI of 0.1 FFU/cell for 48 h. Plates were stained with anti-flavivirus antibody with nuclear counterstain, and percent infection was calculated using the Incucyte automated analysis software. Graphs show the mean ± SD of infection relative to DMSO-treated control wells derived from at least three independent experiments performed in technical duplicate. Best-fit curves were calculated in GraphPad Prism 10. Data in Table 1.

**FIG 2:**
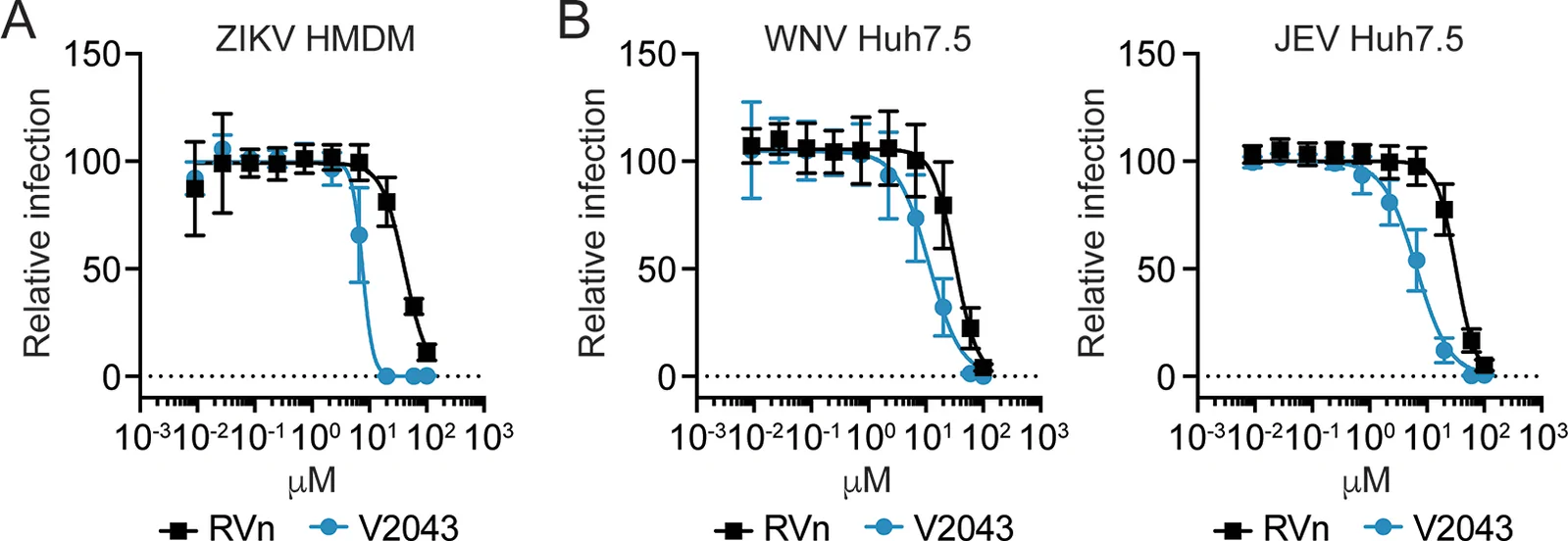
*In vitro* antiviral activity of V2043 and RVn against ZIKV, WNV, and JEV. (A) Primary human monocyte-derived macrophages (HMDMs) were pretreated with compounds for 30–60 min and infected with ZIKV SD001 at an MOI of 1 FFU/cell (based on initial plating density) for 18 h. Virus was pre-incubated 1 h before infection with human-IgG-anti-flavivirus antigen (4G2) to mediate ADE infection. (B) Huh7.5 cells were pretreated with compounds for 30–60 min and infected with WNV NY99 at an MOI of 0.005 PFU/cell for 24 h or JEV genotype III strain 782219 at an MOI of 0.005 FFU/cell for 32–34 h. Plates were stained with anti-flavivirus antibody and nuclear counterstain and imaged on an Incucyte SX5. Percent infection was calculated using the Incucyte automated analysis software. Graphs show the mean ± SD of infection relative to DMSO-treated control wells derived from three to seven independent experiments (at least three with max 100 μM compounds) performed in technical duplicate. Best-fit curves were calculated in GraphPad Prism 10. Data in Table 2.

Myeloid cells, including macrophages and dendritic cells, are among the first cell types to be infected with DENV after inoculation via mosquito bite. Thus, we first compared the antiviral activity of V2043 with RVn against DENV serotype 2 (DENV-2) and ZIKV in primary human monocyte-derived macrophages (HMDMs). We differentiated HMDMs from isolated monocytes and infected them with DENV-2 or ZIKV pre-incubated with non-neutralizing concentrations of anti-envelope antibody (human IgG1–4G2) to enhance infection. V2043 was 8.8× more potent than RVn against DENV and 5.3× more potent than RVn against ZIKV (Tables 1 and 2; Fig. 1A and 2A). To determine if V2043 exhibits antiviral efficacy against all DENV serotypes and other related flaviviruses, we tested V2043 and RVn against DENV serotypes 1 (DENV-1), 3 (DENV-3), and 4 (DENV-4) as well as related flaviviruses West Nile virus (WNV) and Japanese encephalitis virus (JEV) (Fig. 1B and 2B). V2043 showed similar antiviral potency against DENV serotypes 1–4, demonstrating pan-serotype activity. Additionally, the EC_50_s of V2043 were lower than RVn for all dengue serotypes, WNV, and JEV (Tables 1 and 2). These data demonstrate that V2043 has pan-serotype DENV activity and is a more potent broad-spectrum flavivirus inhibitor than RVn.

### V2043 reduces peak serum viral loads in dengue-infected mice

**FIG 3:**
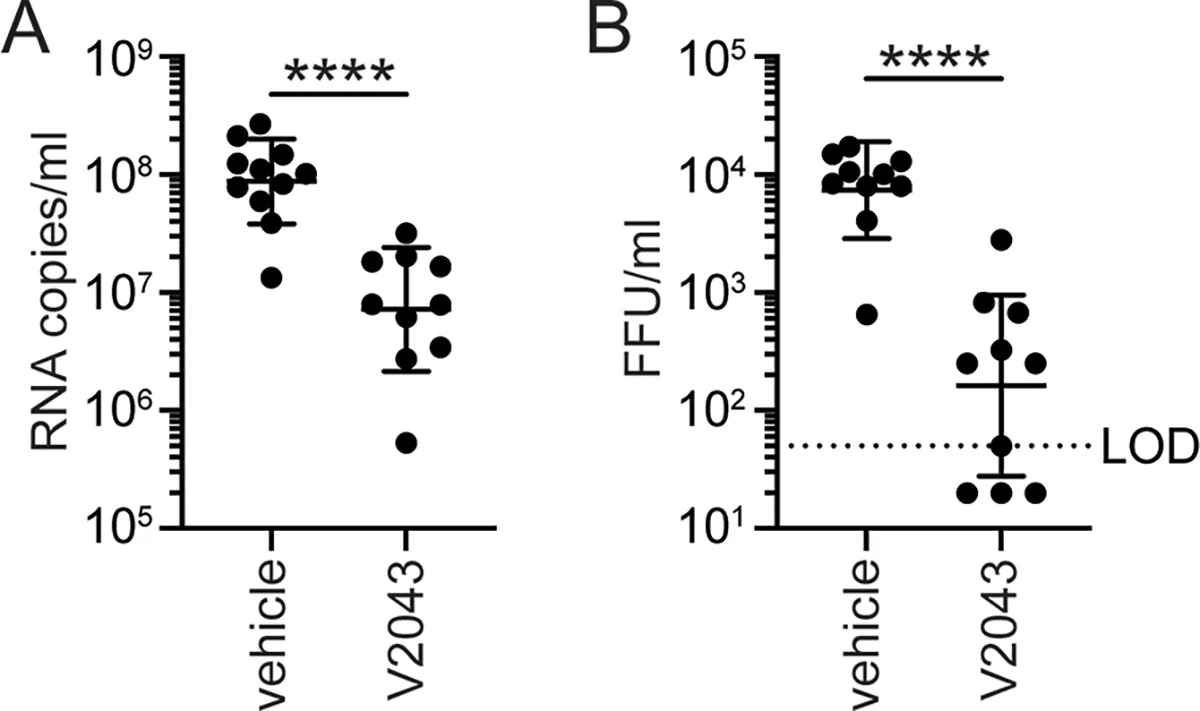
Effect of V2043 treatment on serum viral loads in DENV-infected AG129 mice. AG129 mice (5–6 week-old, male and female) were infected intraperitoneally with 10^6^ FFU of DENV-2 S221 and treated orally once daily with vehicle or V2043 (160 mg/kg loading dose, then daily doses of 80 mg/kg) beginning at the time of infection. Serum was collected at 3 days post-infection. (A) RNA was extracted from serum from *n* = 11 vehicle-treated and *n* = 10 V2043-treated mice, and copies/ml of DENV genome were measured by qRT-PCR. (B) Infectious DENV-2 was quantified by fluorescent focus assay on VeroE6 cells directly after serum collection for *n* = 10 mice per group. Graphs show mean ± SD of log-transformed values compared by unpaired t-test with Welch’s correction. **** =*P* < 0.0001. Graphs show combined results of two experiments. Dotted line in B indicates limit of detection of 50 FFU/ml. Values below LOD were assigned the arbitrary value 20 FFU/ml. One data point was excluded because mouse received an incomplete dose.

AG129 mice lacking IFNα/β and γ receptors are highly susceptible to DENV, and when infected using ADE, recapitulate key features of severe dengue disease in humans, including vascular permeability and increased vasoactive cytokines (13, 14). To measure effects of oral V2043 treatment on peak viremia (day three post-infection) *in vivo*, AG129 mice were infected with 10^6^ FFU of DENV-2 S221 via the intraperitoneal route and treated with V2043 or vehicle by oral gavage beginning at the time of infection. Treatment consisted of one oral loading dose of 160 mg/kg followed by daily doses of 80 mg/kg for 2 days. Viral RNA copies and infectious titers were measured in serum collected on day three post-infection. V2043 significantly reduced serum dengue virus RNA by 1.09 log_10_ and infectious virions by 1.67 log_10_ (Fig. 3A and B). These data demonstrate that V2043 exhibits antiviral activity against dengue virus *in vivo*.

### Prophylactic V2043 prevents the development of severe dengue disease and mortality

**FIG 4:**
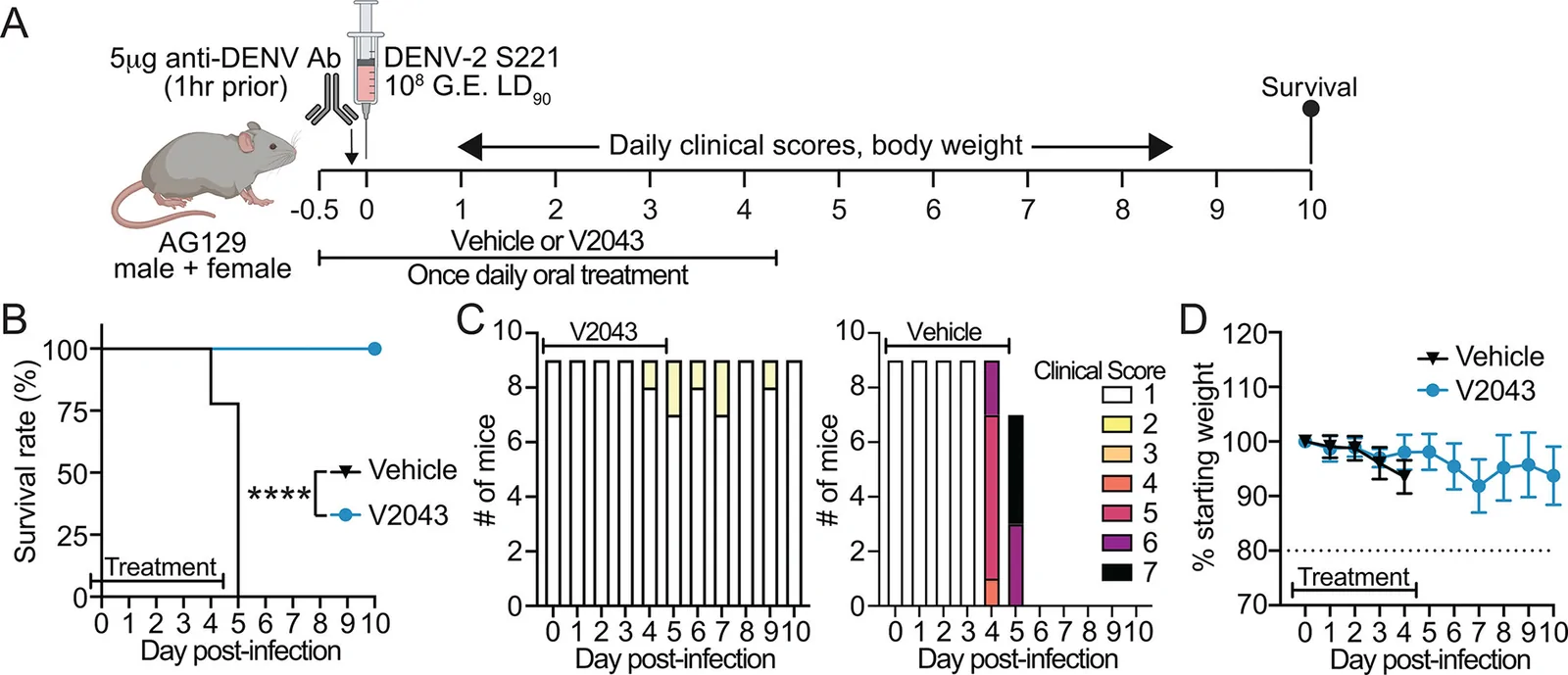
Prophylactic efficacy of oral V2043 in a lethal DENV mouse model. AG129 mice (5–6-week-old, *n* = 9 per group, male and female) were infected with 10^8^ GE DENV2 + ADE. (A) Mice were treated daily beginning 12 h before infection with V2043 (160 mg/kg loading dose, then 80 mg/kg once-daily) or vehicle. Dosing and clinical scoring were blinded. Figure partially created in BioRender (15). (B) Kaplan-Meier survival plots using log-rank (Mantel–Cox) test. **** =*P* < 0.0001. (C) Clinical scores: 1 = healthy; 2 = slightly ruffled (slightly ruffled coat around head and neck, active, alert); 3 = ruffled (ruffled coat throughout body, active, alert); 4 = sick (very ruffled coat, slightly closed/inset eyes, walking but no scurrying, mildly lethargic); 5 = very sick (very ruffled coat, closed/inset eyes, slow to no movement, extremely lethargic); 6 = euthanize (very ruffled coat, closed/inset eyes, moribund or loss of 20% body weight); 7 = deceased. (D) % body weight change from baseline (mean ± SD).

To evaluate the ability of V2043 to protect mice from disease and mortality during lethal DENV infection, mice were intravenously infected with 10^8^ genomic equivalents (GE) of DENV-2 S221 one hour after IP injection of 5 μg anti-DENV antibody (clone 2H2) to induce ADE. Oral treatments of an initial loading dose of 160 mg/kg 12 hours prior to infection followed by five daily doses of 80 mg/kg were administered (Fig. 4A), and body weight, clinical scores, and survival were monitored until 10 days post-infection. Vehicle-treated mice experienced signs of inflammatory disease by day 4 (diarrhea, piloerection, and coat ruffling, extreme lethargy) and death or euthanasia at pre-established humane endpoints by days 4–5 (Fig. 4B). V2043 prophylaxis completely protected mice from severe disease and death (Fig. 4B). Treated mice displayed no or very mild signs of infection, such as mildly ruffled coat and mildly reduced activity (Fig. 4C and D). Thus, prophylactic V2043 treatment is protective against severe dengue disease and mortality *in vivo*.

### Therapeutic V2043 reduces the development of severe dengue disease and mortality

**FIG 5:**
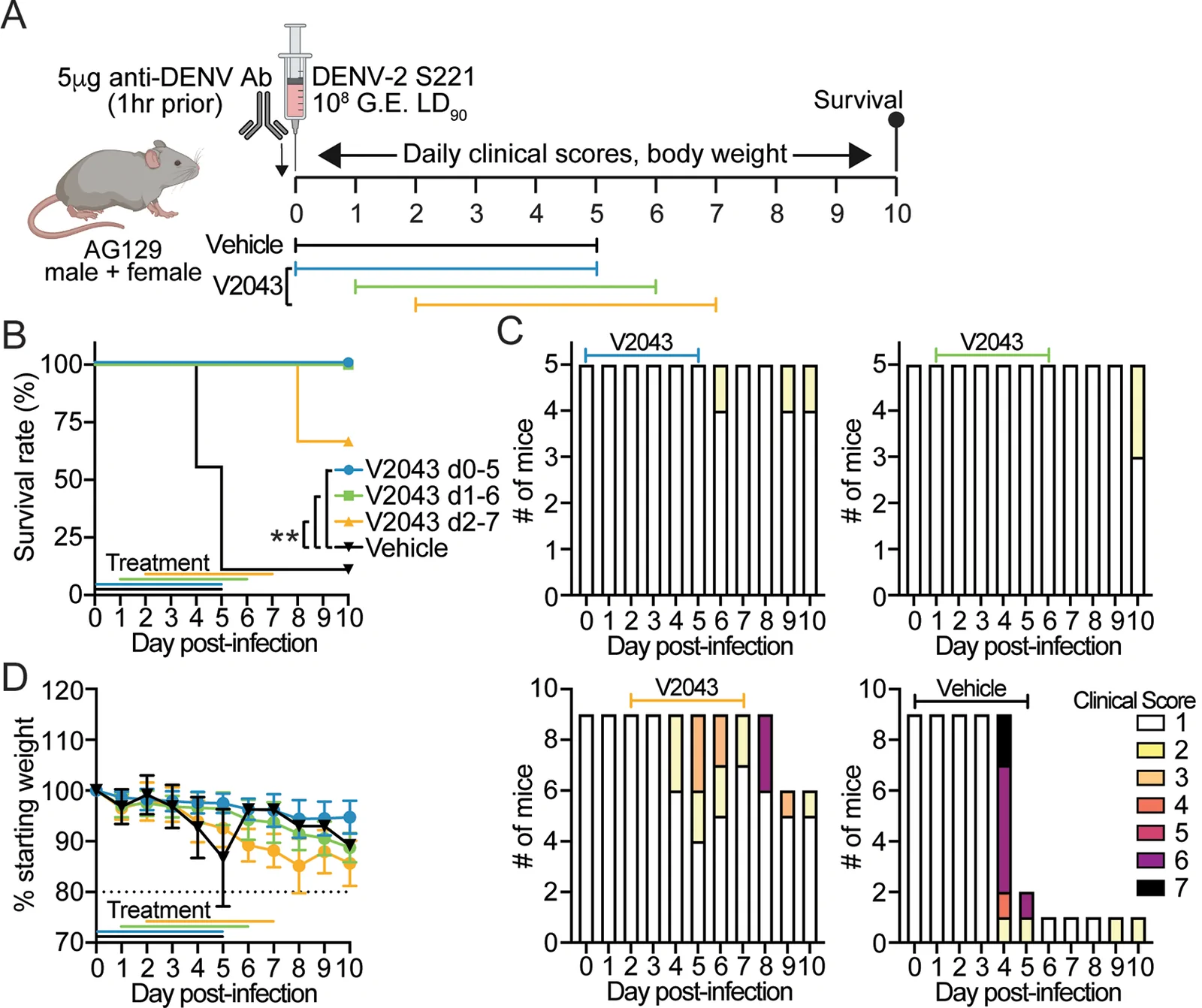
Therapeutic efficacy of oral V2043 in a lethal DENV mouse model. AG129 mice (5–6-week-old, male and female) were infected with 10^8^ GE DENV-2 + ADE. (A) Mice were treated orally with vehicle (*N* = 9) or V2043 (160 mg/kg loading dose, then 80 mg/kg once daily) beginning at the time of infection (*n* = 5), at day 1 (*N* = 5), or at day 2 (*N* = 9) post-infection. Clinical scoring was blinded. Figure partially created in BioRender (15). (B) Kaplan-Meier plots using log-rank (Mantel–Cox) test. (C) Clinical scores: 1 = healthy; 2 = slightly ruffled (slightly ruffled coat around head and neck, active, alert); 3 = ruffled (ruffled coat throughout body, active, alert); 4 = sick (very ruffled coat, slightly closed/inset eyes, walking but no scurrying, mildly lethargic); 5 = very sick (very ruffled coat, closed/inset eyes, slow to no movement, extremely lethargic); 6 = euthanize (very ruffled coat, closed/inset eyes, moribund or loss of 20% body weight); 7 = deceased. (D) % body weight change from baseline (mean ± SD). Graphs show combined results of two experiments.

Next, therapeutic treatment models were applied to determine the *in vivo* efficacy of V2043 administered at the time of or following infection with DENV. AG129 mice infected intravenously with DENV-2 S221 plus ADE were treated with oral V2043 beginning at the time of infection or after a delay of 1 or 2 days. Survival, weights, and clinical scores were measured until day 10 post-infection as in Fig. 5. Treatment with V2043 initiated at the time of infection or one day post-infection completely protected against severe disease and death (Fig. 5B and C). When initial treatment was delayed until 2 days post-infection, V2043 treatment increased survival and reduced disease severity, with all mice surviving until day 8 of 10. Non-survivors displayed only mild signs of disease but met weight loss criteria for humane euthanasia (Fig. 5D).

**FIG 6:**
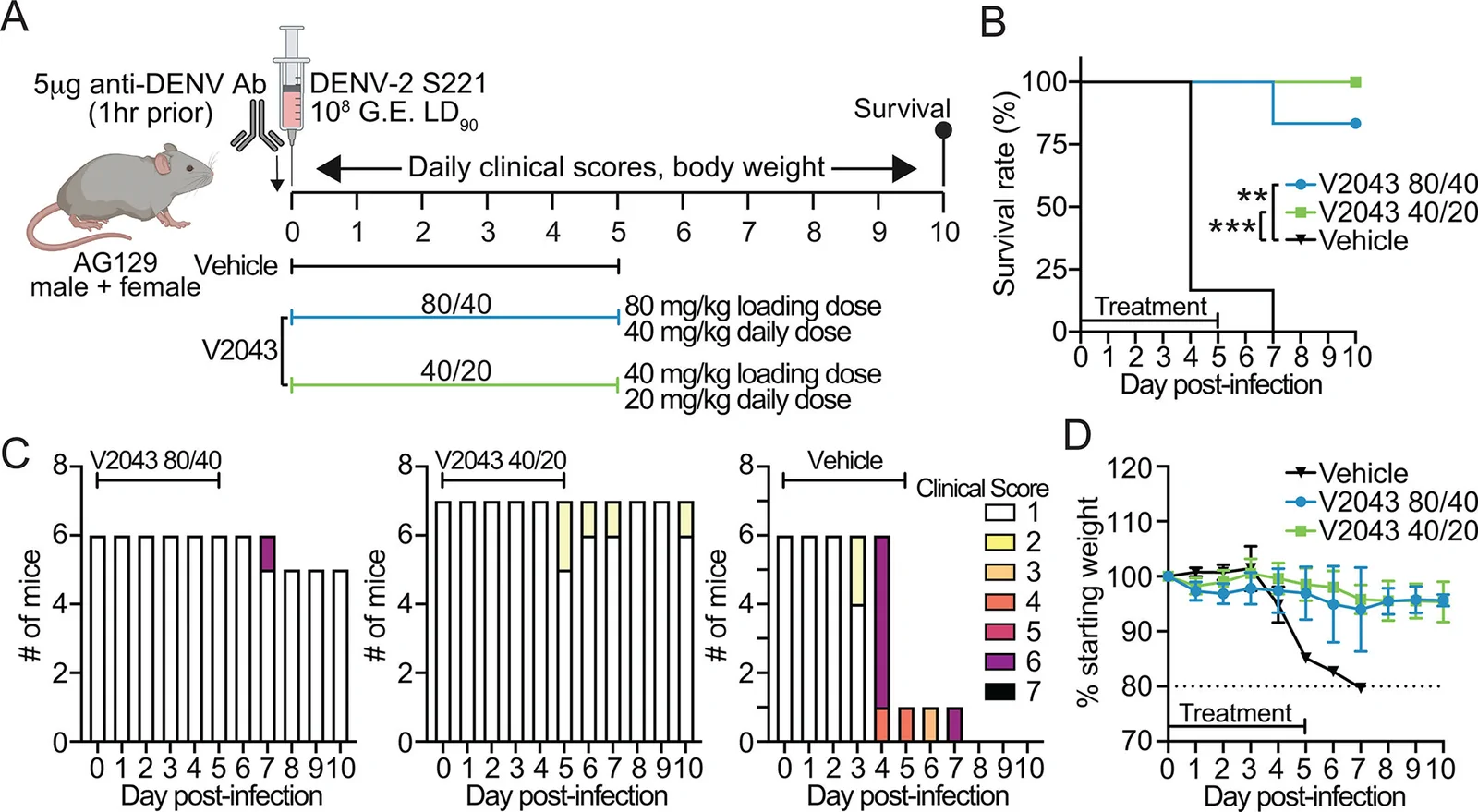
Therapeutic efficacy of reduced-dose oral V2043 in a lethal DENV mouse model. AG129 mice (5–6-week-old, male and female) were infected with 10^8^ GE DENV2 + ADE. (A) Mice were treated orally with vehicle (*N* = 6) or V2043 daily beginning at the time of infection with 80 mg/kg loading dose, then five once-daily doses of 40 mg/kg (80/40, *N* = 6) or with 40 mg/kg loading dose, then five once-daily doses of 20 mg/kg (40/20, *N* = 7). Figure partially created in BioRender (15). (B) Kaplan-Meier plots using log-rank (Mantel–Cox) test. Clinical scoring was blinded. (C) Clinical scores: 1 = healthy; 2 = slightly ruffled (slightly ruffled coat around head and neck, active, alert); 3 = ruffled (ruffled coat throughout body, active, alert); 4 = sick (very ruffled coat, slightly closed/inset eyes, walking but no scurrying, mildly lethargic); 5 = very sick (very ruffled coat, closed/inset eyes, slow to no movement, extremely lethargic); 6 = euthanize (very ruffled coat, closed/inset eyes, moribund or loss of 20% body weight); 7 = deceased. (D) % body weight change from baseline (mean ± SD). Graphs show combined results of two experiments.

To better determine the lowest efficacious dose of V2043, we infected AG129 mice with DENV-2 S221 using ADE infection and treated with 50% (80mg/kg loading dose then 40 mg/kg daily (80/40)) or 25% (40mg/kg loading dose then 20 mg/kg daily (40/20)) of the previously tested dose beginning at the time of infection and followed until 10 days post-infection (Fig. 6A). Both treatment groups exhibited a significant reduction in morbidity and mortality, with 40 mg/kg loading +20 mg/kg daily dose achieving 100% protection against severe disease and death (Fig. 6B–D). Altogether, these data demonstrate that therapeutic V2043 reduces severe dengue disease and mortality *in vivo* and can be effective at low molar doses.

### V2043 is well tolerated when administered daily for 14 days

**FIG 7:**
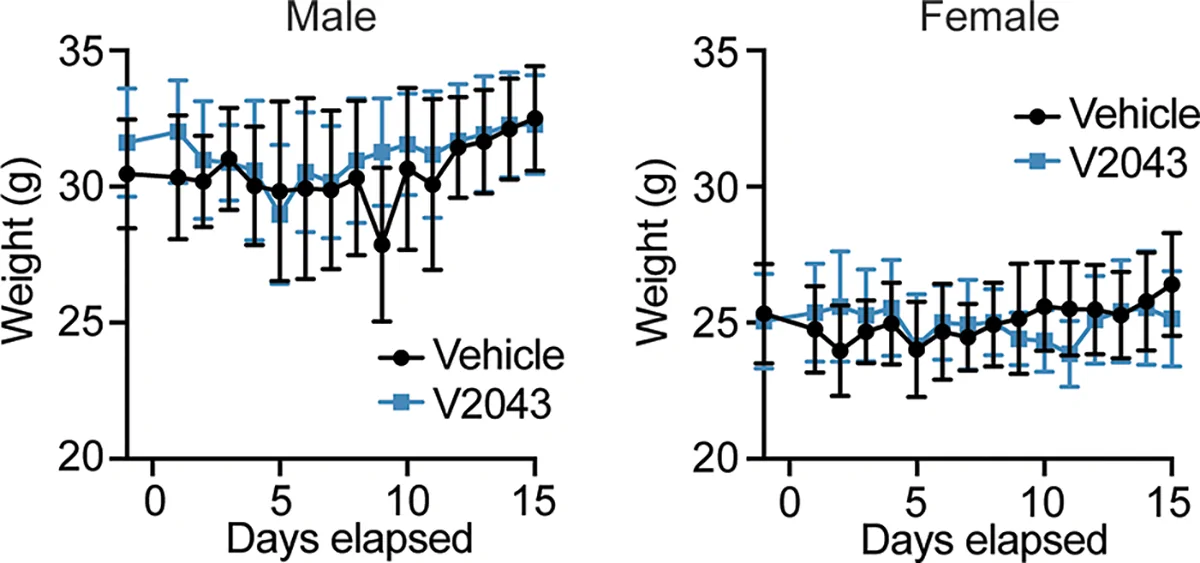
Tolerability of daily oral dosing of V2043 over 14 days. V2043 or vehicle was administered to CD-1 mice by oral gavage at 80 mg/kg/day for 14 days. Graphs show mean ± SD of body weight for *N* = 5 mice per group.

Finally, we performed a 14-day study to assess the tolerability of daily oral V2043 administration. V2043 was administered at 80 mg/kg once daily by oral gavage to male and female CD-1 mice for 14 consecutive days. Body weights were measured and signs of toxicity were assessed daily. At the end of the study, all mice were euthanized. Organs were evaluated by macroscopic pathologic exam, and serum chemistry, hematology, liver function, and lipid analysis were performed. Mice that were administered oral V2043 at 80 mg/kg for 14 days had similar clinical, pathologic, and serum lab values compared to vehicle treatment in both male and female mice, suggesting that oral V2043 was well tolerated over this time period (Table 3; Fig. 7).

## DISCUSSION

There is a great need for broad-spectrum antiviral therapies for the treatment of emerging and contemporary RNA viruses, including DENV. Remdesivir nucleoside triphosphate has broad-spectrum antiviral activity, and an oral prodrug that produces high tissue levels of RVn-TP would enable early outpatient treatment of multiple RNA viruses that lack current treatment options. In this study, we demonstrated that the phospholipid prodrug V2043 provides excellent protection from severe dengue virus disease in mice. Our results showed that V2043 has pan-serotype dengue activity and is a more potent inhibitor of multiple flaviviruses than RVn. *In vivo*, we showed that once-daily dosing with as low as 20 mg/kg after a 40 mg/kg loading dose protected mice from lethal dengue challenge and that the higher dose of 80 mg/kg after a 160 mg/kg loading dose was protective even if treatment was delayed. Finally, we showed that extended treatment with 80 mg/kg of V2043 is well tolerated in male and female mice.

We believe that V2043 has advantages over existing anti-dengue virus drug candidates. Multiple clinical anti-dengue candidates targeting NS4B, including NITD-688 (16, 17), JNJ-A07 (18, 19), and JNJ-1802 (mosnodenvir) (20), show potential as specific treatments for all serotypes of dengue. While we did not directly compare *in vivo* efficacies in this study, V2043 treatment of DENV-2-infected AG129 mice achieved a comparable reduction in viral loads compared to DENV NS4B-targeting compounds, with the significant benefit of possessing broad-spectrum activity against multiple RNA virus families. Additionally, NS4B-directed antivirals can select for viral resistance (16, 18), including in human infections (20), and DENV isolates with known resistance mutations are already circulating (21). Resistance to V2043 may be less problematic due to the high barrier to resistance of its active metabolite RVn-TP. While evidence for RVn-TP resistance in dengue virus is not described to our knowledge, the flavivirus tick-borne encephalitis virus (TBEV) showed only a small reduction in EC_50_, from 0.55 to 1.32 μM, after serial passaging in the presence of remdesivir (22). Although treatment-emergent substitutions in the SARS-CoV-2 RNA-dependent RNA polymerase have been described after remdesivir treatment, primarily in immunosuppressed patients (23–25), resistance to remdesivir has not become widespread after years of use, and multiple phase III trials did not find evidence that remdesivir treatment was associated with emergence of resistance mutations (26, 27). Thus, V2043 treatment in individuals with dengue may be less likely to select for viral resistance than current NS4B-targeting compounds.

Oral RVn prodrugs are in development, including obeldesivir, which successfully achieved secondary endpoints in clinical trials against SARS-CoV-2 when administered twice daily (28). In contrast to V2043, obeldesivir is metabolized during GI absorption to RVn. RVn uptake and initial phosphorylation are relatively slow, limiting its antiviral potency. In contrast, the phospholipid prodrug design of V2043 enhances cellular entry and bypasses initial phosphorylation, which together increase antiviral activity (10). Consistent with this idea, our data demonstrate that V2043 is more potent compared to RVn against all DENV serotypes, ZIKV, WNV, and JEV. Additionally, in contrast to obeldesivir, V2043 achieves high sustained plasma levels after a single daily oral dose (10). This suggests that the potency and pharmacokinetics of V2043 could enable effective treatment of flaviviruses using lower doses and less frequent administration than other RVn prodrugs. Accordingly, we previously found that on a molar basis, once-daily oral V2043 was substantially more active than twice-daily obeldesivir in an *in vivo* mouse model of SARS-CoV-2 (10).

Our study has limitations. First, a side-by-side comparison to other candidate therapies, such as NITD-688, JNJ-A07, JNJ-1802 (mosnodenvir), or obeldesivir, was not performed, and therefore, we cannot make direct comparisons between efficacies of these compounds and V2043. Second, although our infection model recapitulates key aspects of severe dengue disease in humans, there are important differences. We use a high viral inoculum delivered by intraperitoneal or intravenous routes rather than a small intradermal inoculum, which would more closely mimic vector-acquired dengue. Additionally, we utilize ADE and intravenous infection to better recapitulate human pathology associated with severe DENV disease. Thus, the timing of the development of severe disease in mice differs from humans. In mice, signs of disease appear 24–48 h before death or severe disease requiring euthanasia. In contrast, the critical phase in human infection typically occurs 3–7 days after symptom onset. Although we show that V2043 is protective against severe DENV disease in mice when administered approximately 2–3 days prior to mortality, given these differences in disease kinetics, it is difficult to predict the time-dependent efficacy of V2043 in humans. Despite these limitations, we believe that our stringent DENV mouse model creates a high threshold to claim therapeutic efficacy. Collectively, we believe that these encouraging preclinical efficacy and tolerability results strongly support further development of V2043 toward human trials.

## MATERIALS AND METHODS

### Study design

The goal of the study was to evaluate the antiviral efficacy and tolerability of our previously published orally bioavailable prodrug of remdesivir nucleoside, V2043 (ODBG-P-RVn, 1-*O*-octadecyl-2-*O*-benzyl-*sn*-glyceryl-P-RVn), against dengue virus in mice and additional flaviviruses *in vitro*. V2043 was tested against DENV serotypes 1–4 and other flaviviruses in *in vitro* assays with at least *n* = 3 independent experiments conducted in technical duplicate (two wells per condition). *In vivo* studies involving DENV-2 infection were performed after review and approval by the Institutional Animal Care and Use Committee (IACUC) of UC San Diego. Tolerability studies were reviewed by the IACUC of Attentive Science.

Mice were allocated to groups as they became available through breeding. Groups were allocated prior to treatment assignment to equalize sex and litter across groups. Researchers were blinded to treatment assignment during infection, clinical scoring, sample collection, and dosing as was feasible (indicated in figure legends). Researchers were blinded to sample ID during RNA extraction, qRT-PCR, and counting of infectious foci for viral load quantification, and the order of treatments was varied between replicate experiments. Experimental endpoints (survival and day-3 viral loads) were pre-determined.

Sample size (at least *n* = 9 mice per group) was used for viral load and prophylactic treatment experiments. Subsequent experiments used at least *n* = 5 mice per group. *In vivo* survival experiments terminated at 10 days post-infection or upon an animal reaching pre-determined humane endpoints, as approved by UC San Diego IACUC protocol number S18169. Animals included in the study were 5- to 6-week-old males and females. Animals were excluded from the data for improper dose (one animal), for not recovering after anesthesia on day 0 (one animal), or for insufficient sample volume for viral load determination (one animal). Data combined from multiple independent experiments are indicated in the figure legends.

### Mice

AG129 mice were bred and housed in the ABSL2+ facility at UC San Diego on a 12-h light/dark cycle with nesting material and unrestricted food and water access.

### *In vivo* infections

Five- to six-week-old male and female mice weighing at least 16 g on infection day were used for experiments. Mice were infected as described previously (12) with some modifications. When antibody-dependent enhancement (ADE) was included, mice were infected intravenously 1 hour after intraperitoneal injection of 5 μg anti-DENV antibody (2H2) in a volume of 200 μL sterile DPBS. For intravenous infection, mice were anesthetized with isoflurane using a VetFlo precision vaporizer (Kent Scientific), and 10^8^ GE of DENV-2 S221 was injected retro-orbitally in a volume of 100 μL PBS + 10% heat-inactivated FBS. For intraperitoneal infection without ADE, 10^6^ FFU of DENV-2 S221 was injected in 100 μL PBS + 10% heat-inactivated FBS. Mice were weighed with a portable electronic balance at the time of the first dose for prophylactic infection model or at the time of infection and once daily thereafter. Clinical scoring using criteria approved by IACUC was performed blinded to treatment group as follows: 1 = healthy; 2 = slightly ruffled (slightly ruffled coat around head and neck, active, and alert); 3 = ruffled (ruffled coat throughout body, active, and alert); 4 = sick (very ruffled coat, slightly closed/inset eyes, and walking but no scurrying, mildly lethargic); 5 = very sick (very ruffled coat, closed/inset eyes, slow to no movement, and extremely lethargic); 6 = euthanize (very ruffled coat, closed/inset eyes, and moribund or loss of 20% body weight); and 7 = deceased.

### Cells and reagents

Huh7.5 cells (APATH, LLC) were maintained in DMEM (Corning, cat. #10–013-CV-1) with 10% FBS (Biowest, cat. #S1620), 1× Penicillin/Streptomycin (pen/strep, Thermo Fisher Scientific, cat. #15140122). C6/36 (ATCC) were maintained in Leibovitz’s L-15 Medium (Thermo Fisher Scientific, cat. #11415064) with 10% FBS, 1× GlutaMAX (Thermo Fisher Scientific, cat. #35050061), 1× non-essential amino acids (NEAA, Thermo Fisher Scientific, cat. #11140050), 10 mM HEPES (Thermo Fisher Scientific, cat. #15630080), and 1× pen/strep. VeroE6 (ATCC) were maintained in DMEM with 10% FBS and 1× pen/strep. Human monocyte-derived macrophages were differentiated from monocytes isolated from healthy donors as described previously (29, 30). Briefly, blood was collected under UCSD IRB #181624 and centrifuged over Histopaque. Monocytes were purified from isolated buffy coats with the Pan Monocyte Isolation Kit (Miltenyi Biotec, cat. #130–096-537) according to the manufacturer’s instructions. Monocytes were seeded at 80,000 cells per well in 96-well plates and differentiated to macrophages for 7 days in RPMI 1640 (Thermo Fisher Scientific, cat. #11875093) with 10% heat-inactivated FBS, 1× pen/strep, and 1× NEAA with freshly added 100 μg/mL recombinant human macrophage colony-stimulating factor (rh-MCSF, Peprotech, cat. #300–25 or GeminiBio, cat. #300–842P). Medium was refreshed every 3–4 days.

### Viruses

Mouse-adapted DENV-2 S221 was kindly gifted by Sujan Shresta. DENV-2 UIS 353 was acquired from the World Reference Center for Emerging Viruses and Arboviruses. DENV-1 UIS 998, DENV-3 US/BID-V1043/2006, and DENV-4 UIS 497 were acquired from BEI as part of a dengue contemporary virus panel (cat. #NR-51131). WNV 385–99 (NY99) was acquired from BEI (cat. #NR-158). JEV genotype III strain 782219 was acquired from BEI (cat. #NR-2328). ZIKV SD001 (31) was isolated at UC San Diego from a traveler returning to California from Venezuela during the 2016 Zika epidemic.

### Viral propagation

Viruses were inoculated on 70–80% confluent monolayers of C6/36 cells in C6/36 infection medium (L-15 with 5% FBS, 1× pen/strep, and 10 mM HEPES) for 1 h with rocking at 28°C. Following incubation, additional infection medium was added to flasks to a volume of 25 mL per T175 flask. Cells were monitored for CPE, and viral supernatants were clarified by centrifugation at 3,500 RPM for 10 min at 4°C. Virus was purified by overnight 6% polyethylene glycol (PEG) precipitation at 4°C, followed by centrifugation at 10,000 × *g* for 1 h at 4°C. Virus was resuspended in L-15 + 25 mM HEPES, rotated overnight, aliquoted, and stored at −80°C. DENV and ZIKV were titered by fluorescent focus assay on VeroE6 cells. WNV and JEV were titered by plaque assay on BHK-21 cells. DENV-2 S221 genomic equivalents were determined by qRT-PCR using *in vitro* transcribed RNA standard curve as described under qRT-PCR.

### Fluorescent focus assay to quantify infectious virus

VeroE6 cells were seeded at 10,000 cells per well in 96-well plates 1 day before infection. Virus and serum samples were serially diluted 10-fold in DMEM + 1% FBS. Medium was removed from cells, and 30 μL diluted virus was added per well in technical duplicate and incubated with rocking for 1.5 h at 37°C, 5% CO_2_. After adsorption, virus was removed and cells were overlaid with 1% methylcellulose in MEM with 1× pen/strep, 2% heat-inactivated FBS, 1× GlutaMAX, 1× nonessential amino acids, and 10 mM HEPES. Overlay medium was produced by mixing 2× medium (2× MEM, Thermo Fisher Scientific, cat. #11935046 plus the above supplements at 2× final concentration) with 2% (wt/vol) methylcellulose (Millipore Sigma, cat. #M0387), autoclaved in UltraPure water (Thermo Fisher Scientific, cat. #10977023). Assays were incubated 3 days at 37°C, 5% CO_2_. Overlays were removed, and monolayers were washed once with PBS and fixed with 4% formaldehyde in PBS for 30 min, then stained with anti-flavivirus primary antibody (4G2) and AlexaFluor 647 anti-mouse secondary antibody. Foci of infection were counted on images of whole wells acquired at 4× magnification and stitched automatically on an Incucyte SX5 imager (Sartorius).

### qRT-PCR

RNA was extracted from 10 μL sera stabilized in DNA/RNA Shield using the Directzol RNA Microprep kit (Zymo Research) according to the manufacturer’s instructions and eluted in 15 μL. DENV-2 copy numbers were determined by 1-step qRT-PCR on a QuantStudio 5 (Applied Biosystems) with TaqMan Fast Virus 1-Step Mix (Applied Biosystems) using the following primers and probe sequence.

Forward: 5′-AGAGGTGAGGATGGATGCTG-3′, Reverse: 5′-ATGCCATTCCCAAGACTCCT-3′, Probe: 5′-HEX/TTGGTCAAC/ZEN/TCCTTGGTCACAGCC/3′IABkFQ. RNA standard curve was created by adding T7 promoter (underlined) to the forward primer: GAATAATACGACTCACTATAGG-AGAGGTGAGGATGGATGCTG. RNA was *in vitro* transcribed from T7-containing template using mMESSAGE mMACHINE kit (Thermo Fisher Scientific, cat. #AM1344) according to the manufacturer’s instructions. RNA products were purified with the MEGAclear Transcription Clean-up Kit (Thermo Fisher Scientific, cat. #AM1908), visualized by agarose gel electrophoresis, and quantified by Qubit.

### *In vitro* antiviral assays

Antiviral assays were performed as described previously (29). Huh7.5 cells were seeded in 96-well plates at 15–18 × 10^3^ cells/well the day before infection. Cells were pretreated for 60–90 min with serial dilutions of V2043. Virus was added without removing compounds, and cells were incubated 24 h for WNV, 32–34 h for JEV, and 48 h for DENV. Isolated monocytes were seeded in 96-well plates at 80 × 10^3^ cells/well and differentiated to HMDMs as described above. HMDMs were pretreated for 60 min with serial dilutions of V2043 and infected by ADE as described previously (30). Briefly, viruses at MOI = 1 were pre-incubated with human IgG-4G2 anti-envelope antibody in RPMI 1640 with 1% heat-inactivated FBS, 1× pen/strep, 1× NEAA before infection, and 100 ng/mL rh-MCSF. Cells were infected for 1 h, washed once with PBS, and fresh complete macrophage medium with V2043 dilutions was added to cells for 18 h of incubation. Dilutions of V2043 and rh-MCSF were present throughout the assay. Cells were fixed with 4% formaldehyde in PBS and stained with anti-flavivirus envelope (4G2) primary antibody and AlexaFluor 647 anti-mouse secondary antibody with Sytox Green nuclear counterstain. Five images per well were acquired in a pre-set pattern at 10× magnification in an Incucyte SX5 imager. Total nuclei and viral antigen-positive cells were detected by automated Incucyte software analysis and exported as percent-infected cells. Percent infection was normalized to the average of 6–8 DMSO-treated infected wells per plate. Best-fit curves were calculated in GraphPad Prism 10.

### Compounds

V2043 was prepared at Nanosyn (Santa Clara, CA, USA). Synthesis and full characterization of V2043 were described by us previously (7). For oral administration to mice, V2043 was dissolved in sodium bicarbonate/sodium carbonate buffer (pH 9) at concentrations that allowed all mice to receive the same volume (160 μL) of solution by gavage. RVn was purchased from MedChem Express (Monmouth Junction, NJ, USA).

### Statistical analysis

Data pre-processing was performed in Microsoft Excel, and statistical analyses were performed in GraphPad Prism 10. *In vitro* dose-response curves were calculated by non-linear regression. Mean ± SD EC_50_ and EC_90_ values were computed from at least three independent experiments.

## Figures and Tables

**TABLE 1:** *In vitro* antiviral activity of V2043 and RVn against dengue virus^a^

Virus and cell type	Compound	EC50 (μM)	EC90 (μM)	CC50 (μM)	SI	V2043 Rel Act vs RVn

DENV-2 UIS 353 on HMDM	RVn	13.820 ± 7.454	27.592 ± 6.502	>100	>7.2	8.8×
	V2043	1.577 ± 0.927^c^	3.525 ± 0.691	>100	>63.4	
DENV-1 on Huh7.5	RVn	54.500 ± 48.850	–	>100	–	18.1
	V2043	3.017 ± 1.039^b^	10.300 ± 4.216	55.8	18.5	
DENV-2 on Huh7.5^e^	RVn	5.91 ± 2.64	17.12 ± 3.27	>100	>17	6.80
	V2043	1.10 ± 0.27	2.53 ± 1.12	55.8	51.0	
DENV-3 on Huh7.5	RVn	16.390 ± 5.153	46.150 ± 4.440	>100	>6.1	11.7
	V2043	1.400 ± 0.386^d^	4.962 ± 1.785	55.8	39.9	
DENV-4 on Huh7.5	RVn	11.480 ± 3.467	67.730 ± 15.790	>100	>8.7	13.7
	V2043	0.837 ± 0.153^d^	5.772 ± 2.908	55.8	66.7	

a EC_50_, 50% effective inhibition concentration; EC_90_, 90% effective inhibition concentration; CC_50_, 50% cytotoxic concentration; SI, selective index = CC_50_/EC_50_; Rel. Act., Relative Activity based on EC_50_; –, could not be reliably determined at concentrations tested; HMDM, human monocyte-derived macrophage; Huh7.5, human hepatoma cell line. Mean values ± standard deviation values were derived from a minimum of three independent experiments performed in technical duplicate. Log_10_EC_50_ values for V2043 and RVn were compared by paired two-sided *t*-test.

b *P* < 0.05.

c *P* < 0.01.

d *P* < 0.001. CC_50_ values were determined by CellTiter-Glo (max 100 μM). EC_50_, EC_90_, and CC_50_ were calculated in GraphPad Prism 10 software.

e Data included in a previous publication (12).

**TABLE 2:** *In vitro* antiviral activity of V2043 and RVn against ZIKV, WNV, and JEV^ab^

Virus and cell type	Compound	EC50 (μM)	EC90 (μM)	CC50 (μM)	SI	V2043 Rel Act vs RVn

ZIKV SD001 on HMDM	RVn	40.650 ± 6.791	>100	>100	>2.5	5.3×
	V2043	7.644 ± 1.563^e^	13.290 ± 1.455	>100	>13.1	
ZIKV PRVABC59 on Huh7.5^f^	RVn	27.31 ± 0.84	31.85 ± 2.25	>100	>4	>5.1×
	V2043	5.37 ± 1.58	11.46 ± 2.02	55.8	10.0	
WNV on Huh7.5	RVn	29.740 ± 10.120	89.800 ± 17.050	>100	>3.4	2.5×
	V2043	11.700 ± 4.761^c^	40.840 ± 13.760	55.8	4.8	
JEV on Huh7.5	RVn	27.080 ± 1.710	82.830 ± 18.550	>100	>3.7	4.0×
	V2043	6.708 ± 2.223^d^	26.710 ± 8.850	55.8	8.3	

a EC_50_, 50% effective inhibition concentration; EC_90_, 90% effective inhibition concentration; CC_50_, 50% cytotoxic concentration; SI, selective index = CC_50_/EC_50_; Rel. Act., Relative Activity; HMDM, human monocyte-derived macrophage; Huh7.5, human hepatoma cell line. Mean values ± standard deviation values were derived from three to seven independent experiments (at least three with max 100 μM) performed in technical duplicate. Log_10_EC_50_ values for V2043 and RVn were compared by unpaired two-sided *t*-test using at least.

b *P* < 0.05.

c *P* < 0.01.

d *P* < 0.001.

e *P* < 0.0001. CC_50_ values were determined by CellTiter-Glo (max 100 μM). EC_50_, EC_90_, and CC_50_ were calculated in GraphPad Prism 10 software.

f Data included in a previous publication (28).

**TABLE 3:** Hematology, liver function, serum chemistry, and lipid analysis after 14 days of daily oral dosing^a^

	Male vehicle (2)	Male V2043 (3)	Female vehicle (3)	Female V2043 (2)

HGB (g/dL)	14.6 ± 0.4	14.8 ± 0.2	14.4 ± 0.3	15.1 ± 0.1
HCT (%)	46.5 ± 0.2	46.5 ± 0.5	44.5 ± 1.1	47 ± 0.5
PLT (10^3^/μL)	1,078 ± 91	1,246 ± 70	576 ± 411	1,041 ± 130
WBC (10^3^/μL)	6.38 ± 3.26	5.04 ± 0.22	4.77 ± 0.39	5.22 ± 1.2
Retic (%)	2.78 ± 0.53	2.91 ± 0.72	3.37 ± 1.01	3.41 ± 0.15
Phos (mg/dL)	8.2 ± 0.5	9 ± 1.2	8.5 ± 0.7	10.4 ± 2
	Male vehicle (3)	Male V2043 (3)	Female vehicle (3)	Female V2043 (3)

Cholesterol (mg/dL)	148 ± 8	131 ± 8	122 ± 6	122 ± 15
Triglyceride (mg/dL)	223 ± 76	160 ± 30	213 ± 35	104 ± 31^b^
Glucose (mg/dL)	204 ± 12	199 ± 5	210 ± 54	215 ± 13
	Male vehicle (3)	Male V2043 (3)	Female vehicle (3)	Female V2043 (3)

ALT (U/L)	23 ± 5	15 ± 2	21 ± 2	17 ± 3
ALP (U/L)	82 ± 8	94 ± 13	112 ± 27	112 ± 19
AST (U/L)	68 ± 40	44 ± 2	49 ± 3	62 ± 16
TBIL (mg/dL)	0.5 ± 0.1	0.4 ± 0.1	0.4 ± 0.1	0.4 ± 0.2
TP (g/dL)	5.4 ± 0.1	5.3 ± 0.4	5.4 ± 0.3	5 ± 0.4
ALB (g/dL)	3.1 ± 0.1	3 ± 0.2	3.1 ± 0.2	2.9 ± 0.1
	Male vehicle (3)	Male V2043 (3)	Female vehicle (3)	Female V2043 (3)

BUN (mg/dL)	22 ± 3	22 ± 1	22 ± 5	14 ± 2
Crea (mg/dL)	0.35 ± 0.02	0.36 ± 0.03	0.39 ± 0.03	0.37 ± 0.07
CK (U/L)	262 ± 204	102 ± 28	95 ± 8	152 ± 68
Ca (mg/dL)	9.8 ± 0.3	10.1 ± 0.1	9.9 ± 0.4	9.8 ± 0.3

a V2043 (80 mg/kg/day) or vehicle was administered to CD-1 mice by oral gavage for 14 days prior to analysis. Mean ± SD values for *n* = 2–3 mice per group (numbers in parentheses) and V2043-treated and vehicle-treated mice compared by unpaired *t*-tests independently based on sex.

b *P* < 0.05.

## Data Availability

All data are available in the main text or the supplemental material.
